# Comparison of Peritoneal Adhesion Formation in Bowel Retraction by Cotton Towels Versus the Silicone Lap Pak Device in a Rabbit Model

**Published:** 2011-11-07

**Authors:** Brian G. Liu, Dawn S. Ruben, Wolfgang Renz, Antonio Santillan, Steven J. Kubisen, John W. Harmon

**Affiliations:** ^a^Johns Hopkins University, Baltimore, MD; ^b^Johns Hopkins University, School of Medicine, Baltimore, MD; ^c^McGill University, Montreal, Canada; ^d^Cancer Care Centers of South Texas, San Antonio, TX; ^e^Seguro Surgical, Columbia, MD; ^f^Johns Hopkins University, Bayview Medical Center, Baltimore, MD

## Abstract

**Objective:** Manipulation of cotton operating room towels within the abdominal cavity in open abdominal surgery has been associated with the formation of peritoneal adhesions. In a rabbit model, the use of standard cotton operating room towels is compared to the Lap Pak, a silicone bowel-packing device, to determine the potential for reducing the risk of adhesions. **Methods:** Thirty rabbits were randomly assigned to 3 groups. The rabbits underwent a sham surgery with incision only (n = 10), placement of operating room towels (n = 10), or placement of a Lap Pak (n = 10). After 14 days, the rabbits were sacrificed and the peritoneal cavity explored for adhesions. The number, tenacity, ease of dissection, and density of adhesions were recorded, and the adhesions quantitatively graded using a Modified Hopkins Adhesion scoring system. **Results:** The operating room towel group had an average adhesion score of 2.5, and 8 (80%) rabbits developed adhesions. The sham group had an average adhesion score of 0.3 and one rabbit (10%) developed adhesions. The Lap Pak group had an average adhesion score of 0.2 and 1 rabbit (10%) developed adhesions. The frequency and severity of adhesions in the operating room towel group were significantly greater from that of the baseline sham group. There was no significant difference between the Lap Pak and sham groups. **Conclusions:** In this rabbit laparotomy model, the use of the Lap Pak to retract the bowels resulted in significantly fewer adhesions compared to cotton operating room towels. Lap Pak may be beneficial for bowel packing in general abdominal surgeries.

Postoperative adhesions (POAs), the formation of abnormal fibrous attachments between tissues after procedures such as laparotomies represent a significant source of morbidity and mortality following abdominal surgery. In addition to patient safety concerns, POAs place a significant economic burden on US healthcare. It is estimated that up to 93% of patients undergoing a laparotomy will develop abdominal adhesions,^1^^-^3 which can result in a variety of sequelae including chronic abdominal pain, small bowel obstruction, and female infertility, with a lifelong risk for reoperation to ameliorate such complications.^4^^-^7 More than 33% of patients undergoing abdominal surgery will have at least one hospital admission in the 10 years following surgery to treat adhesions associated with their initial surgery.^8^^,^^9^ In women, the problem is particularly severe. Postoperative adhesions occur in up to 90% of women undergoing major gynecological surgery, with estimates suggesting that up to 20% of cases of infertility are secondary to adhesions.^10^^,^^11^ Each year, in the United States alone, more than 400,000 adhesiolysis operations are performed to treat POA complications, costing the healthcare system approximately $2 billion.^12^ The incidence of these complications has risen over the past decade, with recent estimates for the annual cost of treating bowel obstructions due to adhesions reaching $3.45 billion.^13^

Lap Pak (Fig 1) is an FDA (Food and Drug Administration) registered surgical device designed to reduce trauma to the bowels during abdominal surgery by reducing bowel manipulations, distributing pressure more evenly throughout the cavity, and maintaining a constant temperature and humidity within the bowels. The one-piece device interfaces with a standard retractor apparatus used in laparotomies, as shown in the second panel of Figure 1, and can also be used in laparoscopic surgeries involving a hand-assisted incision or gel port. In addition to the features mentioned earlier, Lap Pak is composed of silicone, an inert and biocompatible material, and has smooth surfaces and rounded edges to minimize trauma to the serosal surface of the bowels. It is nonabsorbent and will preserve the moisture of the bowels, rather than leaching fluids.

The current commonly used bowel retracting method is to use cotton towels or sponges to assist with retraction during routine abdominal surgeries. Several studies have shown that the abrasive texture of cotton and the remaining fibers left behind in the cavity contribute to the formation of abdominal adhesions.^13^^-^16 In particular, manipulation of cotton operating room (OR) towels during surgery introduces abrasions to the serosa, acting as a significant source of adhesions.^17^^-^19 Because the towels do not retain their position well, readjustment throughout the surgery is necessary to maintain exposure of the surgical site. The Lap Pak was chosen for comparison to cotton materials because it eliminates the repacking process used with standard cotton towels and sponges. There are 3 important design features, seen in the top panel of Figure 1. First, the lower flange projects forward to prevent the Lap Pak from flipping out of position. In addition, the large soft wings encompass the bowels to hold them in place. Finally, the central segment of the Lap Pak is relatively stiff, allowing it to maintain its position even when pressure is applied from the retractors.

The aim of this study is to compare 2 methods of bowel retraction: insertion and manipulation of cotton materials within the cavity as in current standard practice, and the application of the silicone Lap Pak without the need for manipulation. Using a rabbit laparotomy model, we compare outcomes on a macroscopic level by examining the presence of postoperative adhesions using a rabbit laparotomy model for the 2 methods. We hypothesize that the Lap Pak will significantly reduce the incidence of postoperative adhesions compared to the use of cotton towels.

## MATERIALS AND METHODS

This study was approved by the Johns Hopkins University Institutional Animal Care and Use Committee and is in compliance with the *Guide for the Care and Use of Laboratory Animals*^20^ and the Animal Welfare Act. Thirty male New Zealand White rabbits (Myrtle's Rabbitry, Thompsons Station, Tennessee) weighing from 3 to 4 kg were used. These rabbits were from a colony negative for cilia-associated respiratory bacillus, *Encephalitozoon cuniculi*, *Pasteurella multocida*, and *Treponema cuniculi*. The rabbits were housed individually in a room with a 12-hour light cycle, temperature 21°C to 22 °C and relative humidity 50% to 70%. The rabbits were fed free choice with commercially available high-fiber pellets (High Fiber Rabbit Diet 2031, Harlan, Frederick, MD) and reverse-osmosis water provided via an automatic watering system. Each rabbit received timothy hay (Johns Hopkins Farm, Baltimore, MD) weekly for enrichment.

For each surgical procedure, the surgeon wore sterile, powder-free, Biogel PI gloves (Molnlycke Health Care, Norcross, GA) to reduce the risk of any adhesions due to the type of surgical glove used. The abdomen was shaved and prepped with povidone iodine and isopropyl alcohol. An Ioban drape was placed over the abdomen to prevent any contamination from stray rabbit hairs or debris in the air. Each rabbit was anesthetized with ketamine (40 mg/kg) and acepromazine (2 mg/kg) intramuscularly, intubated, and maintained on isoflurane. An indwelling catheter was placed in an ear vein for administration of intraoperative 0.9% saline. As preemptive analgesia, the rabbits received buprenorphine (0.01 mg/kg) intramuscular. Once the rabbit was anesthetized to a surgical plane of anesthesia, a 4 to 6 cm caudal ventral midline incision was performed. Each rabbit was randomly assigned to one of 3 groups: sham surgery, Lap Pak, or standard OR towels. We chose to simulate bowel packing consistent with major colorectal or OB-GYN surgery.

## LAPAROTOMY ADHESION MODEL

### Sham group

For the 10 sham rabbits, no manipulation of the bowels was performed after the abdominal incision. A paper drape was placed over the open incision to help prevent further heat loss and any airborne contamination. The rabbit was maintained under anesthesia for four hours, consistent with the average length of time for a major colorectal or OB-GYN surgical procedure.

### Lap Pak group

For the 10 Lap Pak (Seguro Surgical LLC, Columbia, Maryland) rabbits, a 6-inch by 4-inch section of one of the Lap Pak wings was cut and placed in the abdomen since the Lap Pak in its entirety was too large to be placed into the rabbit's abdomen. The cut edge was not placed into the abdomen but was left exposed through the incision to prevent the rough cut edge from increasing the risk of adhesion formation. A paper drape was placed over the open incision to help prevent further heat loss and any airborne contamination. The Lap Pak was left in place for 4 hours.

### Operating room towel group

The final 10 rabbits were assigned to the cotton OR towel group. A standard lint-free cotton OR towel was moistened with 0.9% irrigation saline. The towel was then placed into the caudal abdomen to separate the urinary bladder from the GI tract. Manual manipulation was performed using 2 gloved fingers for 2 minutes to simulate initial bowel packing in a human surgery. A paper drape was placed over the open incision to help prevent further heat loss and any airborne contamination. At the end of each hour, the paper drape was lifted, the towel was removed, remoistened, replaced into the abdomen and manual manipulation was performed for 2 minutes to simulate repacking. This was continued for 4 hours.

### End of procedure and follow-up

After 4 hours, the paper drape cover was removed. Either the Lap Pak or OR towel was removed. The abdominal muscle and skin incisions were closed with 3-0 absorbable braided sutures in a continuous pattern for the muscle and a subcuticular pattern for the skin. Fourteen days (± 30 hours) following surgery, the rabbits were euthanized and a necropsy was performed to evaluate the abdominal contents and record any adhesions. Adhesions were scored using a Modified Hopkins Adhesion Score (Table 1).^21^ Frequency of adhesions, tenacity, gross density, and ease of dissection from surrounding organs were determined. In the scoring system, the most severe variable is used to determine the score. For example, a rabbit with 4 adhesions that are below 100 g in tenacity will still grade as a 4 due to frequency, while a rabbit with 1 adhesion with tenacity above 1000 g will also grade as a 4 due to tenacity. To determine tenacity, a digital scale (American Weigh Scales SR-1KG Digital Hanging Scale, Norcross, Georgia) was used. The scale hook was placed under the adhesion and the organs on either side were held in place. The monitor was then pulled and the grams required to tear the adhesion from tissue was recorded.

### Statistical analysis

Statistical analysis was performed using the Statistical Package for Social Sciences (SPSS, Version 19.0, Chicago, Illinois) software. The nonparametric Mann-Whitney *U* test was used to compare the frequency of adhesions and adhesion grade between groups. Significance level was set at *P* < .05.

## RESULTS

### Sham group

In the sham group, 9 rabbits were free of adhesions, and 1 rabbit (10%) had 1 adhesion that formed between the colon and the incision. The adhesion had a tenacity of 802 g and an overall adhesion score of 3. The rabbits that did not form adhesions were considered to have adhesion score 0. The average number of adhesions per rabbit was 0.1, and the average adhesion score was 0.3.

### Lap Pak group

**Figure 2:**
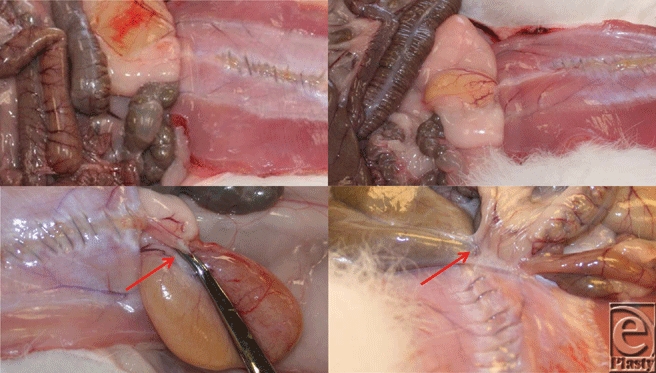
Necropsies of Sham, OR, and LP Rabbits. Photographs taken on postoperative day 14. Starting from the top left and going clockwise: sham rabbit, free of adhesions; Lap Pak rabbit, free of adhesion; OR towel rabbit, adhesion between colon and small intestines, small intestines and incision, colon to incision; OR towel rabbit, adhesion between incision and bladder. Red arrows indicate adhesions; Photographs are cropped from original size.

In the Lap Pak group, 9 rabbits were free of adhesions, and 1 rabbit (10%) had 2 adhesions, both formed between the jejunum and the incision. The adhesions had tenacities of 109 g and 12 g for an overall adhesion score of 2. For the rabbit with adhesions, the Lap Pak was repositioned several times before achieving proper placement. The average number of adhesions per rabbit was 0.2, and the average adhesion score was 0.2. Images of adhesion-free cavities are shown in Figure 2.

### OR towel group

In the OR towel group, 8 rabbits (80%) developed a total of 20 adhesions. The number of adhesions in a single rabbit for this group ranged from 0 to 4, and the average number of adhesions per rabbit was 2. The tenacity of these adhesions ranged from 24 g to over 1090 g (maximum of the scale), and the adhesion scores ranged from 0 to 4, with an average adhesion score of 2.5. The most common adhesions were jejunum to incision (8), colon to incision (6), and urinary bladder to incision (5). The remaining adhesion was a multiorgan adhesion involving the jejunum, colon, and bladder all adhered to each other and to the incision. Images of adhesions are shown in Figure 2.

### Statistical analysis

**Figure 3:**
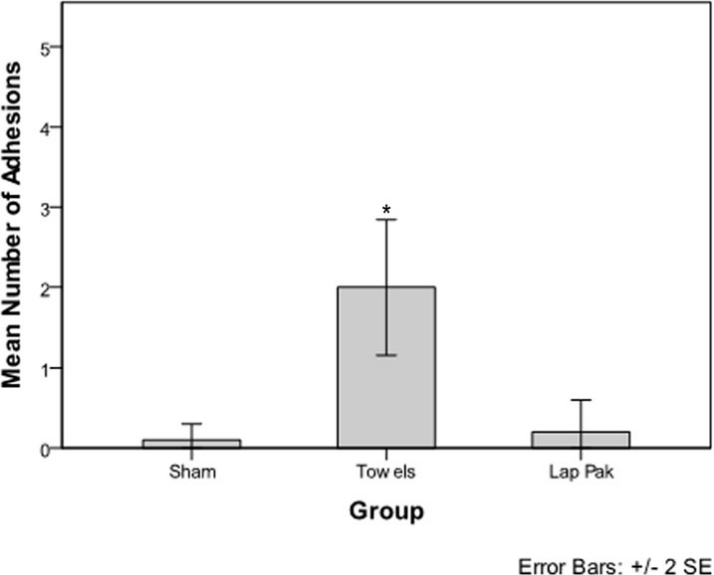
Frequency of Adhesions. Mean number of adhesions per rabbit for the sham surgery, OR towel, and Lap Pak groups. Adhesions were manually counted on postoperative day 14. The asterisk indicates significance at. *P* < .05.

**Figure 4:**
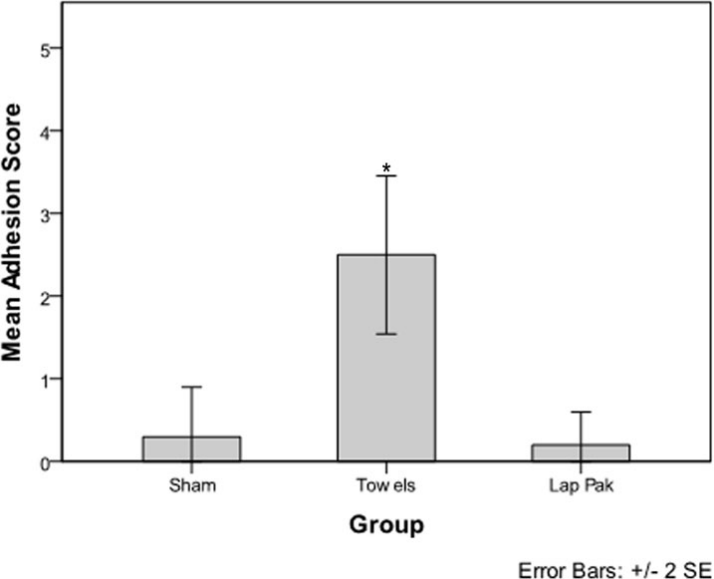
Adhesion Grades. Mean adhesion score per rabbit for the sham surgery, OR towel, and Lap Pak groups. Adhesions were manually inspected using dissection instruments and a digital scale hook. Adhesion score was determined using a Modified Hopkins Adhesion Score. The asterisk indicates significance at. *P* < 0.05.

The OR towel group had a statistically significant higher number of adhesions per rabbit (*P* = .002) compared to the sham group, while the Lap Pak group did not have a significantly higher number of adhesions per rabbit compared to the sham group (Fig 3). Rabbits in the OR towel group had a statistically significant higher adhesion score compared to the sham group (*P* = 0.005), while there was no significant difference in adhesion score between rabbits in the Lap Pak and sham groups (Fig 4).

## DISCUSSION

Prevention of adhesion formation following major surgery continues to be an unmet need that poses a real problem in terms of quality of life and healthcare costs. Resolving the issue of adhesion formation would reduce readmission rates, numbers of additional corrective procedures, and patient distress, all of which are incentives for patients, hospitals.^22^

In this experimental model, application of the Lap Pak, a silicone bowel retracting device, was associated with significantly fewer postoperative adhesions compared to application of cotton OR towels following laparotomy. The sham group provided a baseline level of adhesion formation, which was not significantly different from the Lap Pak group (Fig 3). The use of cotton OR towels was associated with frequent, dense, and strong adhesions, most likely due to cotton fibers abrading serosal surfaces.^16^^,^^19^ In the sham group, one rabbit developed adhesions despite no manipulation of the bowels. This indicates that the causes of adhesion formation are multifactorial and more research is required to determine other underlying causes. For the single rabbit in the Lap Pak group that had 2 adhesions, it is possible that the repositioning of the Lap Pak contributed to the adhesions to form, suggesting that the Lap Pak best reduces adhesions when applied with a reasonable skill level. Although the scope of this study does not allow us to characterize the underlying mechanisms by which adhesions form, the data showed a strong association between manipulation of cotton towels during surgery and adhesion formation. For this study, we limit our conclusions to physiological factors on a macroscopic level, specifically manipulation and application of cotton towels and the Lap Pak. For future studies, histologic examination of the tissues and characterization of the biochemical response to tissue trauma would be useful for understanding the mechanisms by which adhesions form.

**Figure 5:**
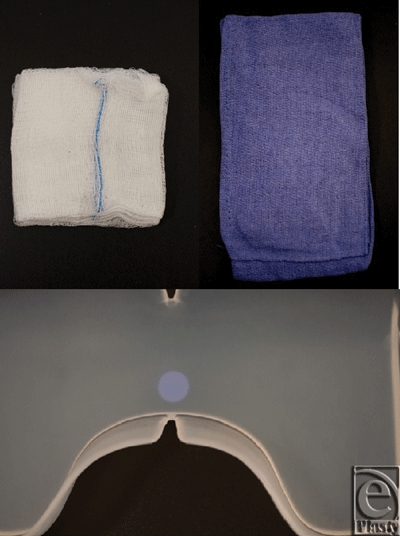
Close-up photographs of cotton and silicone materials. *Top left*: Cotton laparotomy sponges. *Top right*: Cotton operating room towels. *Bottom*: Silicone Lap Pak.

Furthermore, we hypothesize that the difference in outcomes may also be due to the difference in surface texture between cotton and silicone. As seen in Fig 5, the cotton sponges and towels have rough textures compared to the silicone Lap Pak, which as a smooth surface. Of note, a screen-like pattern was frequently observed after removal of cotton materials, possibly indicating physical trauma. However, while the literature does show that physical trauma to the bowels is contributory to adhesions, further studies will be required to directly link the difference in outcome to the surface properties of Lap Pak and cotton towels. Specifically, microscopic observation of tissues postsurgery could be performed in the future to determine this.

In addition to studying adhesion prevention, this study introduces a method for standardizing adhesion testing. While previous studies^23^^-^25 use largely qualitative data points to grade adhesions, the Modified Hopkins Adhesion Score uses the number of adhesions and tenacity in grams, neither of which are subject to observer bias. This allows for more uniform grading and more accurate comparison between the test groups. We also hypothesize that tenacity can also be used as a predictor of morbidity; that is, adhesions that require more force to separate will cause more acute symptoms. Further studies to correlate this measure of adhesion tenacity with severity of symptoms would be of interest to afford an even better understanding of the complex and very important area of postoperative adhesions.

This study highlights potential postoperative complications that can result from the current standard of care for bowel packing in laparotomies. While numerous studies have shown the effect of OR towels and accompanying abrasion from manipulation in packing of bowels,^13^^-^18^,^^26^^,^^27^ this is the first study (to the best of our knowledge) that presents an alternative device and method and its impact on postoperative adhesions in a rabbit model. Bowel dehydration, bowel temperature fluctuation, and trauma from bowel manipulation are also associated with the current bowel packing procedure. These effects are also known to be associated with post operative ileus,^28^^-^30 and further studies have been planned to explore whether the Lap Pak design can minimize these effects. A potential future direction for reducing adhesions through both physical and biochemical means would be to incorporate a drug delivery platform, such as a surface coat of anti-inflammatory or antiproliferative agents. The device could also be modified for laparoscopic procedures and could eventually play a role in both open and minimally invasive surgeries.

Although a clinical trial would be required to compare the clinical outcome of using the Lap Pak and cotton materials, it has been suggested that animal models can provide a reasonable predictor of clinical outcomes. Specifically, the rabbit model has been shown to be useful for adhesion related studies.^31^ For this study, we do not attempt to extend our animal data to clinical outcomes in human subjects. One potential method that we could use for future clinical studies would be noninvasive adhesion detection via abdominal ultrasonography.^32^^,^^33^

Comparison of cotton to a silicone device as an alternate means of bowel packing has not been previously described in the literature. We report the efficacy of Lap Pak in reducing the risk of adhesions in a rabbit model. Lap Pak may be preferable over cotton towels and sponges in cases that require clearing the surgical site by bowel packing. The total implications of the current laparotomy bowel packing methods on post operative complications appears to be an area where further study is warranted, given the large healthcare costs and patient outcomes associated with these complications.

## Acknowledgments

This study was supported in part by the Maryland Technology Development Corporation and US Biotechnology Stimulus Fund.

## Figures and Tables

**Figure 1:**
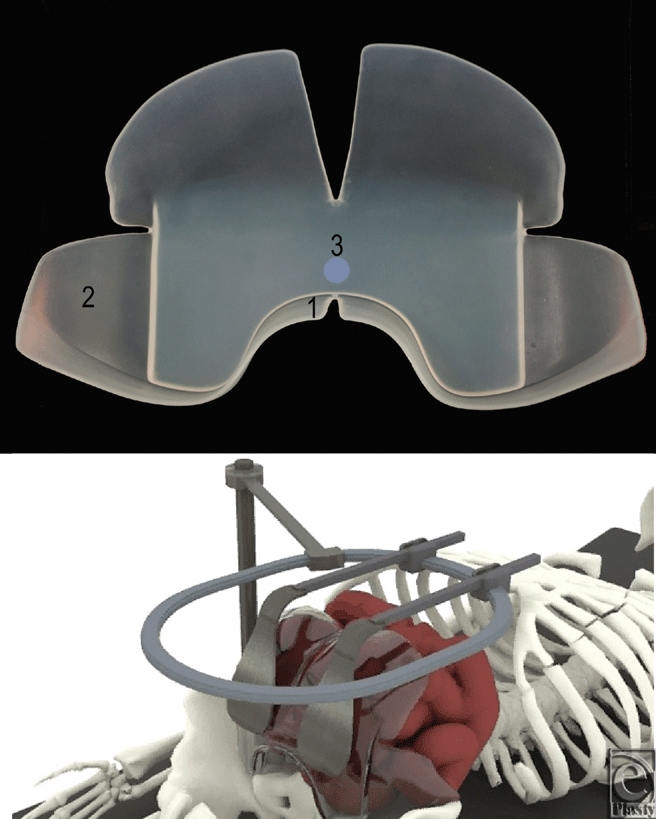
The Lap Pak. *Top panel*: front view of the Lap Pak. The 3 key elements of the Lap Pak are labeled: (1) lower flange, (2) flexible side wings, and (3) thick central body. *Bottom panel*: Computer generated visualization of Lap Pak application in the abdominal cavity.

**Table 1:** Modified Hopkins Adhesion Score*

Score	Frequency	Tenacity (g)	Density	Ease of Dissection
0	0	0	0	No adhesions
1	1	1-100	Thin, transparent	Tears easily
2	2-3	101-500	Semi transparent	Blunt dissection needed
3	3+	501-1000	Opaque, dense	Sharp dissection needed, no serosal damage
4	3+	1001+	Opaque, dense	Sharp dissection needed, serosal damage

*Adhesions are graded on the basis of frequency (number of adhesions in 1 rabbit), tenacity (grams of pressure required to tear the adhesion), density, and ease of dissection.

**Figure:**
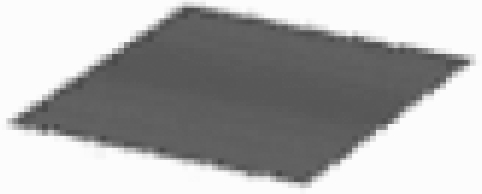
“This video is in QuickTime format. If you do not have QuickTime you may download it here”.
